# The nematode worm *Caenorhabditis elegans* as an animal experiment replacement for assessing the virulence of different *Salmonella enterica* strains

**DOI:** 10.3389/fmicb.2023.1188679

**Published:** 2023-06-08

**Authors:** Wiebke Burkhardt, Carina Salzinger, Jennie Fischer, Burkhard Malorny, Matthias Fischer, Istvan Szabo

**Affiliations:** Department Biological Safety, German Federal Institute for Risk Assessment, Berlin, Germany

**Keywords:** *Caenorhabditis elegans*, *Salmonella*, survival assay, virulence, animal experiments, replace, reduce

## Abstract

*Caenorhabditis* (*C*.) *elegans* has become a popular toxicological and biological test organism in the last two decades. Furthermore, the role of *C. elegans* as an alternative for replacing or reducing animal experiments is continuously discussed and investigated. In the current study, we investigated whether *C. elegans* survival assays can help in determining differences in the virulence of *Salmonella enterica* strains and to what extent *C. elegans* assays could replace animal experiments for this purpose. We focused on three currently discussed examples where we compared the longevity of *C. elegans* when fed (i) with *S*. *enterica* serovar Enteritidis vaccination or wild-type strains, (ii) with lipopolysaccharide (LPS) deficient rough or LPS forming smooth *S*. *enterica* serovar Enteritidis, and (iii) with an *S. enterica* subsp. *diarizonae* strain in the presence or absence of the typical pSASd plasmid encoding a bundle of putative virulence factors. We found that the *C. elegans* survival assay could indicate differences in the longevity of *C. elegans* when fed with the compared strain pairs to a certain extent. Putatively higher virulent *S. enterica* strains reduced the lifespan of *C. elegans* to a greater extent than putatively less virulent strains. The *C. elegans* survival assay is an effective and relatively easy method for classifying the virulence of different bacterial isolates *in vivo*, but it has some limitations. The assay cannot replace animal experiments designed to determine differences in the virulence of *Salmonella enterica* strains. Instead, we recommend using the described method for pre-screening bacterial strains of interest to select the most promising candidates for further animal experiments. The *C. elegans* assay possesses the potential to reduce the number of animal experiments. Further development of the *C. elegans* assay in conjunction with omics technologies, such as transcriptomics, could refine results relating to the estimation of the virulent potential of test organisms.

## 1. Introduction

In the past 20 years, the nematode worm *Caenorhabditis* (*C*.) *elegans* has become one of the most widely used model organisms for nearly every aspect of toxicology and biology (Leung et al., 2008; Meneely et al., 2019). In toxicology, for example, *C. elegans* involves determining the possible harmful effects of chemicals. Although animal tests have traditionally been the backbone of toxicology, currently, a broad range of *in vitro* test methods are available, such as cell cultures, organoids, organs-on-chip, and *in silico* systems (Calonie et al., 2022). The bigger challenge is replacing animal tests in the investigations of more complex biological systems such as the immune system, the circulatory system, or the nervous system. Invertebrate animals offer an alternative to the usual animal tests in mammals for research fields like genetics, physiology, biochemistry, evolution, and neurobiology (Singkum et al., 2019). *C. elegans* is used to study various biological processes, including apoptosis, cell signaling, cell cycle, cell polarity, gene regulation, metabolism, aging, and sex determination (Kaletta and Hengartner, 2006). Infection biology is one of the most complex fields of biological and medical research, and infection models are influenced by a broad range of variables in the host, such as the immune system, the unspecific infection defense, the entrance tissue, and the general immune status (Bulitta et al., 2019). Invertebrate animals such as nematodes or insects provide an *in vivo* research platform that is more complex than cell cultures or organoids. Moreover, these systems are not linked to the ethical and animal welfare concerns that limit the use of the usual animal models (Singkum et al., 2019).

When *C. elegans* was established at the beginning of the 21^st^ century as a model host for studying the pathogenesis of *Pseudomonas aeruginosa*, it was expected that the model would be limited to pathogens with a broad host range. However, a restriction was observed, especially for intracellular pathogen microorganisms such as *Salmonella* and *Listeria*. Nevertheless, at the same time, *Sarratia marcescens* was reported as a second bacterium that was pathogenic to *C. elegans* (Kurz and Ewbank, 2000). Since *C. elegans* came to be used as an infection model, two different effects of *Pseudomonas aeruginosa* have been observed in the nematode. The “fast killing” effect with nematodes dying within 24 h and the “slow killing” effect where the worms survived over several days. The “fast killing” effect was caused by a bacterial toxin, while the “slow killing” effect was seen as an infection-like process (Finlay, 1999). In 2000, the application of the *C. elegans* infection model to *Salmonella* was described for several *Salmonella enterica* serovars, including the serovar Typhimurium (Aballay et al., 2000; Labrousse et al., 2000). In contrast to *Pseudomonas aeruginosa, Salmonella enterica* serotype Typhimurium colonized the intestine of the worm permanently (Aballay and Ausubel, 2002). In 2006, Kaletta and Hengartner regarded *C. elegans* as a system that still needed to prove whether it could be used as a valid and relevant infectious disease model (Kaletta and Hengartner, 2006). In the meantime, *C. elegans* as an infection model has provided many insights into the underlying mechanisms of human diseases, including biological processes such as defensive host response to microorganisms, pathogenic mechanisms, and symbiotic interactions (Kumar et al., 2020).

The role of *C. elegans* as an alternative to animal experiments is being continuously discussed and investigated. The range of applications of the model is extensive and includes nutritional studies on probiotic host interactions, immunity, and infection and studies on the antimicrobial effects of food supplements (Lang et al., 2021; Chakravarty, 2022; Zermeño-Ruiz et al., 2022). The application of genetically modified microorganisms could reveal details on the infection mechanisms, e.g., of *S*. Typhimurium, and the host response of *C. elegans*, where several anti-microbiotic protein pathways have been identified to be linked to the reaction of *Salmonella* virulence factors (Tenor et al., 2004; Sahu et al., 2013). Moreover, different mutants of *C. elegans* can be used to investigate pathogen-host interactions (Aballay and Ausubel, 2002). In animal experiments, different species (e.g., mice, fowl, and pigs) are widely used for studying the general and species-related course of infection by the foodborne pathogen *S. enterica*. The application of a broader range of alternative non-animal-based models could contribute to understanding infection mechanisms and reducing the number of animal experiments.

*Salmonella* can be host-restricted, be host-adapted, or have a broad host range, but only a relatively small proportion of the ca. 2,600 described serovars are of significant clinical relevance. Depending on the serovar, ingested dose, and immunocompetence of the host, *Salmonella* infections differ substantially in their clinical manifestations, ranging from an asymptomatic state to severe illness (Simon et al., 2023). Many virulence factors (e.g., *Salmonella* pathogenicity islands, endotoxins, and virulence plasmids) have been shown to play different roles in the pathogenesis of *Salmonella* infections in humans and animals. Among the virulence traits and factors of *S. enterica* are the invasion of and intracellular replication inside the host's cells (Jajere, 2019). In several studies, *Salmonella* Typhimurium was found to be pathogenic to *C. elegans* and can be lethal to the nematode (Aballay and Ausubel, 2002; Sem and Rhem, 2012). However, the pathogenesis of *S*. Typhimurium infection in *C. elegans* has not been fully clarified. Both well-known *Salmonella* virulence factors and aspects that do not involve the classical invasive or intracellular phenotype of the pathogen appear to play a role in the pathogenicity for the nematode. For example, *S*. Typhimurium has been shown to provoke overwhelming systemic oxidative stress in *C. elegans* through the redox activity of bacterial thioredoxin (Sem and Rhem, 2012).

The present study aimed to determine whether a *C. elegans* survival assay could help measure differences in the virulence of *Salmonella enterica* strains and determine to what extent *C. elegans* assays could replace animal experiments for this purpose. We accordingly selected three examples currently discussed in the literature where specific *Salmonella* characteristics play a role in the pathogenicity course of the organism with possible consequences to control measurements when detected in livestock. We compared (i) vaccination and wild-type strains of *S*. *enterica* serovar Enteritidis (hereafter referred to as *S*. Enteritidis) since vaccination plays a vital role in *Salmonella* control programs. However, evidence of vaccine *Salmonella* strains on table eggs is not yet substantial enough to influence foodstuff legislation. We also compared (ii) lipopolysaccharide (LPS) deficient (also known as rough) with functional LPS (also known as smooth) strains of *S*. Enteritidis. The pathogenicity of *Salmonella* is associated with the presence of the immune-reactive O-chain of the LPS expressed on its surface. Several previous studies have indicated the role of LPS in the pathogenicity of the bacteria in host-pathogen interactions with the innate immune system (Maldonado et al., 2016). Finally, we investigated (iii) the impact of the absence of a type IV secretion system (T4SS)-containing plasmid named pSASd on the pathogenicity of *S*. *enterica* subsp. *diarizonae* (hereafter referred to as SASd).

## 2. Methods

### 2.1. Bacterial strains

SASd (strains 12-01777-0-S2 and 12-01777-0-S3), *S*. Enteritidis (20-SA01872-0), and *S*. Enteritidis (20-SA00671-0 and 09-02812-0) (Table 1) were obtained from the National Reference Laboratory (NRL) for *Salmonella* at the Federal Institute for Risk Assessment, Berlin, Germany (BfR) strain collection. The vaccine strain Salmovac SE (19-SA01616) was obtained from the manufacturer.

**Table 1:** Details of the bacterial strains and their characteristics.

**Strain**	**Subspecies**	**Serovar**	**Source**	**Identifier (SRA accession/biosample)**	**Characteristic**	**References**
*Salmonella enterica*	*diarizonae*	61:k:1,5, (7)	Sheep	12-01777-0-S2 (SRR13071109/SAMN16814736)^*^	Carrier of plasmid pSASd	Uelze et al., 2021
			Sheep	12-01777-0-S3 (SRR23581851/SAMN33408750)	Absence of plasmid pSASd	
	*enterica*	Enteritidis	Vaccine, IDT Biologica GmbH	19-SA01616	Vaccine strain Salmovac SE	IDT Biologica GmbH, Germany (CEVA)
			Chicken	20-SA01872-0 (SRR23581848/SAMN33408753)	Non-vaccination strain	This study
			Bird	20-SA00671-0 (SRR23581850/SAMN33408751)	Smooth surface	This study
			Laying hens	09-02812-0 (SRR23581849/SAMN33408752)	Rough surface	Szabo et al., 2017

*Salmonella* isolates have been deposited in the National Center for Biotechnology Information (NCBI) Sequence Read Archive (SRA) under the BioProject accession number PRJNA937468, ^*^Isolate 12-01777-0-S2 under BioProject PRJNA678834.

To minimize potential variations of results based on the genetic diversity of *S*. Enteritidis strains, we chose isolates belonging to MLST type 11 that showed a close genetic relatedness in whole genome sequencing based on cgMLST analysis.

For the *Salmonella* vaccine and wild-type strain comparative analysis (i), we chose an *S*. Enteritidis isolate from the NRL for *Salmonella* strain collection with a genetic distance of 130 allelic differences (AD) from the vaccine strain to ensure a similar genetic background of both isolates.

To compare the rough and smooth *S*. Enteritidis (ii), we chose two serologically different isolates with a genetic difference of 32 AD.

For the SASd strains (iii), we chose two isolates of ST432, one of which contained a 43 kb large plasmid (pSASd having a T4SS and a toxin/antitoxin system) and one without pSASd (Uelze et al., 2021). The AD between the two strains was 39 in the cgMLST analysis.

*Escherichia coli* OP50 was derived from the strain collection of the group Strategies for Toxicological Assessments at the BfR. Bacterial strains were cultured aerobically in 5 ml Luria-Bertani (LB) medium under shaking at 37°C overnight. The next day, the entire volume was poured into a bottle containing 200 ml of fresh LB medium and incubated at 37°C for 8 h. The bacterial cells were washed three times in M9 buffer (Wittkowski et al., 2020) and up-concentrated. The optical density at 600 nm (OD_600_) was measured (Ultrospec 10, Amersham Biosciences, UK), and the suspension was brought to an OD_600_ corresponding to 10^10^ CFU/ml (according to prior growth experiments as described in Wittkowski et al., 2020). This bacterial suspension was used to inoculate nematode growth medium (NGM) agar plates (Wittkowski et al., 2020) overnight at 37°C and subsequently stored at 4°C for later use in the survival assay.

### 2.2. Serotyping

Strains were serotyped by slide agglutination as described in a previous study (Szabo et al., 2017). Compared to smooth isolates, rough isolates showed a non-specific reaction with all sera and a negative reaction or agglutination with 1 x phosphate-buffered saline.

### 2.3. Whole genome sequencing of *S. enterica* strains

Genomic DNA was extracted from liquid cultures using a PureLink genomic DNA mini kit Invitrogen (Carlsbad, CA, USA). Sequencing libraries were prepared with the Nextera DNA Flex library preparation kit Illumina (San Diego, CA, USA) according to the manufacturer's protocol. Paired-end sequencing was performed on an Illumina MiSeq benchtop sequencer using the MiSeq reagent kit v3 (600 cycles). Raw reads were trimmed and *de novo* assembled with the Aquamis pipeline v1.3 (git version is v1.0.0–60-g60e9d09) (https://gitlab.com/bfr_bioinformatics/AQUAMIS) (Deneke et al., 2021a), which implements fastp v0.19.5 (Chen et al., 2018) for trimming and shovill v1.1.0 (https://github.com/tseemann/shovill) for assembly.

Draft genome assemblies were characterized with the BakCharak pipeline v2.0 (git version 1.0.0–77-g5b31a01) (https://gitlab.com/bfr_bioinformatics/bakcharak), and allele distances between the paired isolates were computed with the chewieSnake pipeline v1.2 (Deneke et al., 2021b) as described in a previous study (Uelze et al., 2021).

### 2.4. Nematode strain

The genetically modified strain *C. elegans* SS104 (genotype glp-4(bn2) I.) was provided by the *Caenorhabditis* Genetics Center (CGC), University of Minnesota (USA), which is funded by the NIH Office of Research Infrastructure Programs (P40 OD010440). The nematode was grown at 16°C on NGM agar plates seeded with *E. coli* OP50 as the sole food source and transferred to fresh food plates two times per week (Wittkowski et al., 2020). Genetically modified worms can reproduce at a permissive temperature of 16°C. At higher temperatures of ~25°C, the worms become sterile and thus cannot produce progeny. This allows us to perform survival experiments for several weeks without the bias of new generations.

### 2.5. Survival assay

*C. elegans* SS104 was washed from a food plate with 10 ml M9 buffer and synchronized with 12% bleach and 1M NaOH as described in a previous study (Wittkowski et al., 2020). Eggs were incubated overnight at 20°C in M9 buffer under shaking, and the resulting L1 larvae were seeded on a fresh food plate and incubated at 25°C for 48 h. From the resulting L4 larvae, 15 were individually picked and transferred to an NGM plate (in 22.1 cm^2^ Petri plates) colonized with the bacterial strain of interest (test plate). The two strains of *Salmonella*, which were meant to be compared, were investigated at the same time on individual plates, while test plates seeded with *E. coli* OP50 were run in parallel as a control. All test plates were incubated at 25°C to avoid the reproduction of the thermo-sterile *C. elegans* strain and counted every weekday until all worms were dead. To discriminate dead from living worms, they were gently poked with a worm picker to observe a touch response. For each bacterial strain, three biological and three technical experiments were carried out. Altogether nine replicate experiments were performed, resulting in a total of 135 worms being used per test strain.

### 2.6. Statistics

Survival was calculated per day relative to the initial number of worms and presented as mean ± standard error of mean (SEM). For the visualization and statistical analyses, the software GraphPad Prism v8.2 (GraphPad Software, San Diego, CA, USA) was used. Differences in the area under the curve (AUC, reflecting the total lifetime of all worms in an experiment) and survival rate per day were tested for significance using the unpaired *t*-test. Survival curves per group were compared with the Gehan-Breslow-Wilcoxon test. The *P*-values of <0.05 relative to the control (*E. coli* OP50) or between both isolates tested were considered significantly different.

### 2.7. Sequencing data information

Sequencing data for *Salmonella enterica* isolates originating from the strain collection of the NRL for *Salmonella* used in this study have been deposited in the National Center for Biotechnology Information (NCBI) Sequence Read Archive (SRA) under the BioProject accession numbers PRJNA937468 and PRJNA678834.

## 3. Results

### 3.1. Vaccine strain Salmovac SE was less virulent against *C. elegans* than a wildtype *S*. Enteritidis strain

**Figure 1:**
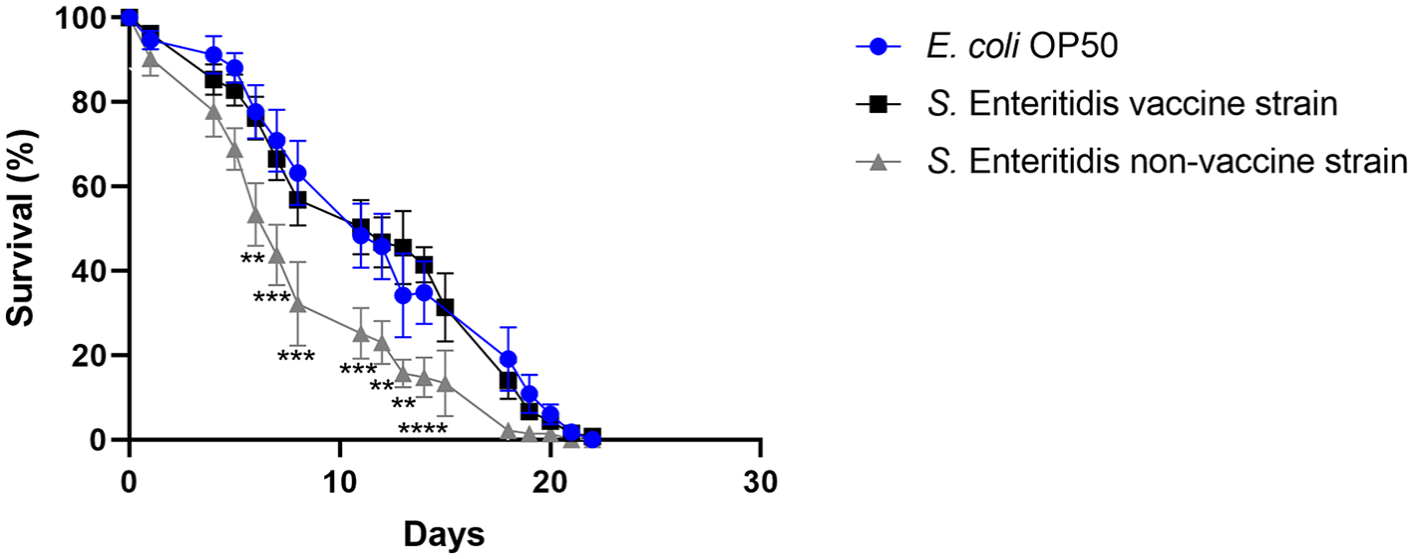
Survival over time of *C. elegans* grown on NGM agar either colonized with *E. coli* OP50 (control), Salmovac SE vaccine strain *S*. Enteritidis 19-SA01616, or non-vaccine strain *S*. Enteritidis 20-SA01872. Mean with SEM, *n* = 9, ^**^p < 0.1, ^***^p < 0.01, ^****^p < 0.001.

**Figure 2:**
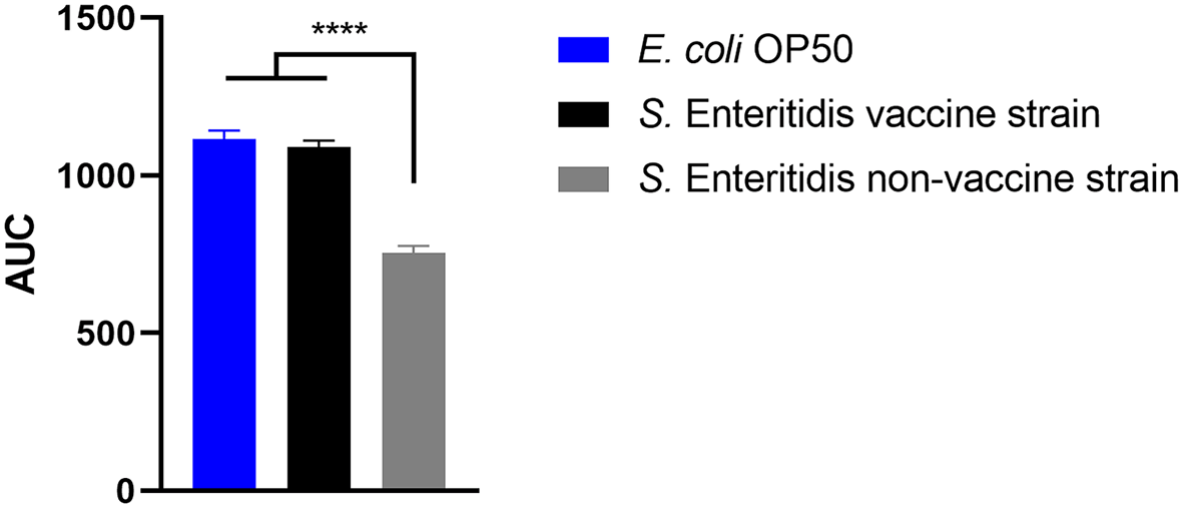
Area under the curve (AUC) of *C*. elegans survival curves. The worms were either fed with control strain *E. coli* OP50, Salmovac SE vaccine strain *S*. Enteritidis 19-SA01616, or non-vaccine strain *S*. Enteritidis 20-SA01872. Mean with SEM, *n* = 9, ^****^p < 0.0001.

The ability of the vaccine strain 19-SA01616 (licensed under the name Salmovac SE) to shorten the lifespan of *C. elegans* was compared to that of the non-vaccination strain *S. enterica* 20-SA01872. Salmovac SE is a live-vaccine auxotrophic for adenine and histidine that was derived through undirected chemical mutagenesis, leading to a significant virulence attenuation (Martin et al., 1996a,b). As shown in Figure 1, there was no difference in the survival rate of *C. elegans* between the vaccine strain and the commensal *E. coli* OP50 (Gehan-Breslow-Wilcoxon test: *p* = 0.3668). In contrast, the non-vaccine strain 20-SA01872 significantly shortened the lifespan of *C. elegans*, especially between days 6 and 14 (Figure 1, Gehan-Breslow-Wilcoxon test: *p* = 0.0008). A comparison of the AUC of the non-vaccination strain with the control group and the vaccine strain revealed a significantly higher difference between them (Figure 2). While the total lifespan of the worms fed with the vaccine strain was reduced by 32%, those of the worms fed with the non-vaccine strain were only reduced by 2% compared to the control group.

### 3.2. The effect of rough or smooth *S*. Enteritidis strains on the longevity of *C. elegans* did not differ

**Figure 3:**
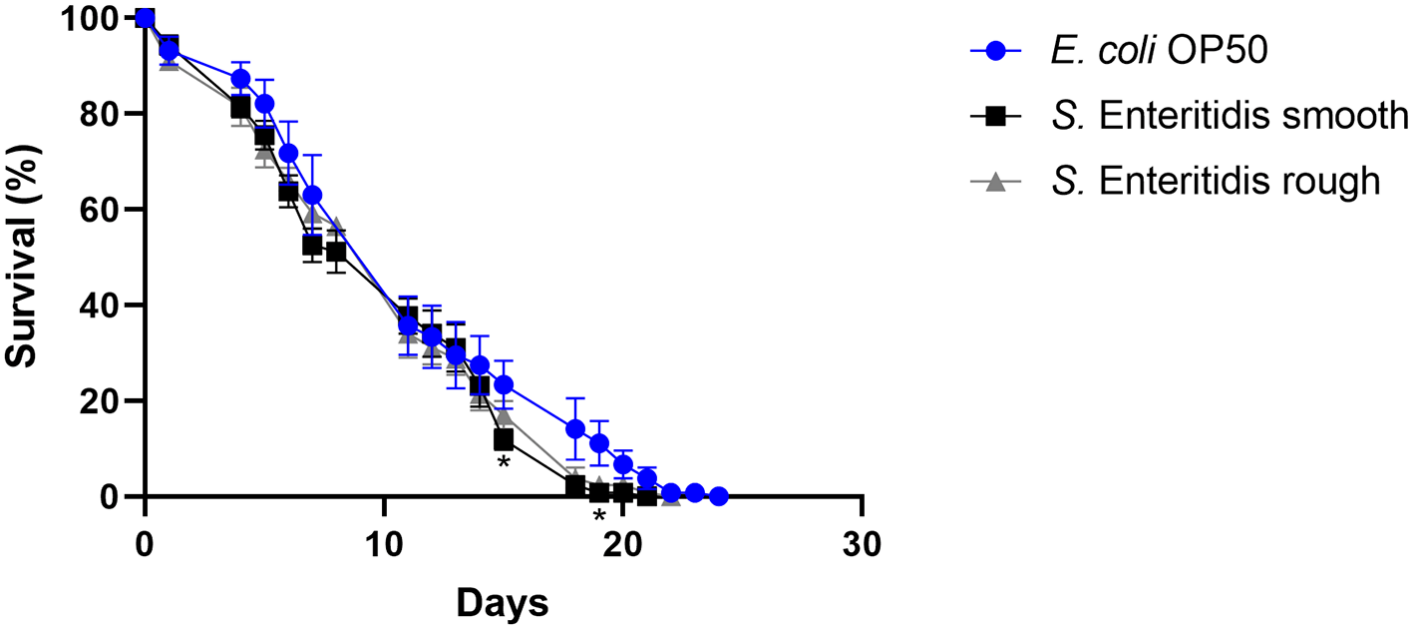
Survival over time of *C. elegans* grown on NGM agar either colonized with *E. col*i OP50, smooth *S*. Enteritidis 20-SA00671, or rough *S*. Enteritidis 09-2812. Mean with SEM, *n* = 9, ^*^p < 0.05.

**Figure 4:**
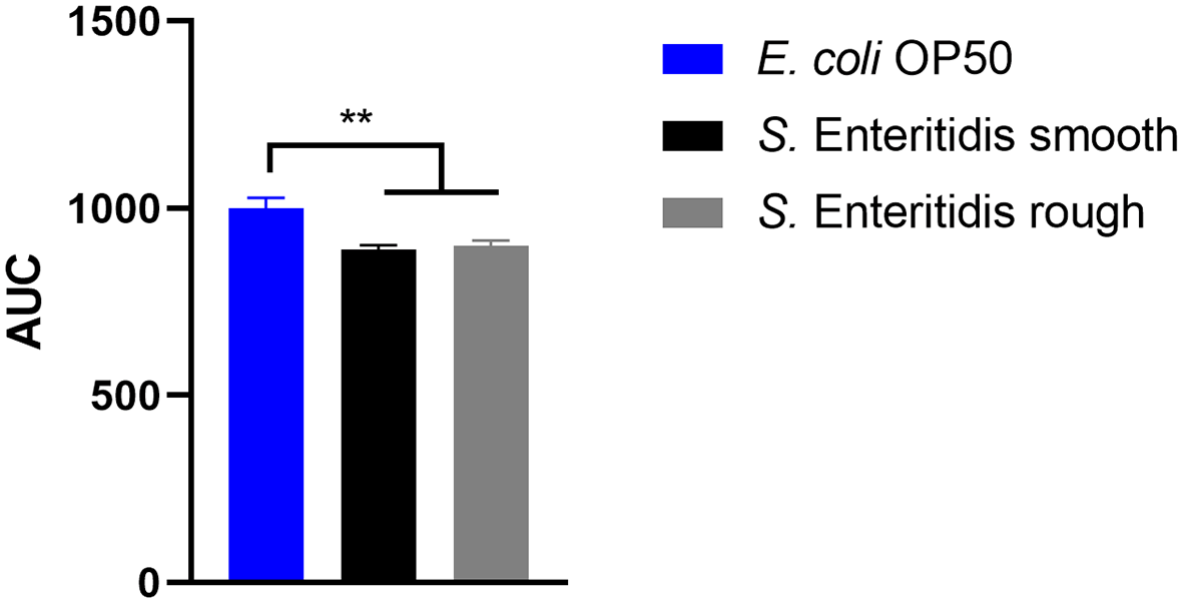
Area under the curve (AUC) of C. *elegans* survival curves. The worms were either fed with *E. coli* OP50 (control), *S*. Enteritidis 20-SA00671 (smooth), or *S*. Enteritidis 09-2812 (rough). Mean with SEM, *n* = 9, ^**^p < 0.01.

When *S*. Enteritidis is found in an environmental, food, or feed sample in the European Union, the measures taken depend on the results of its serotyping (Anonymous, 2011). If not typable, the serotype remains unknown and is referred to as rough (Szabo et al., 2017), and no measures are taken. However, the genetic background might clearly indicate the assignment to *S*. Enteritidis. Therefore, the ability to reduce the lifespan of *C. elegans* was investigated as a surrogate for the pathogenicity of the rough strain 09-2812 and the smooth strain 20-SA00671. As observed in Figures 3, 4, no differences in survival rate were observed for worms fed on the rough or smooth isolate (10 and 11% shorter survival rates compared to the control, respectively). Additionally, differences in virulence were detected between the control *E. coli* strain and the rough and smooth *S. enterica* strains (Figure 4). Overall, the comparison of survival curves revealed a significant difference between the groups in the Gehan-Breslow-Wilcoxon test (*p* = 0.0270).

### 3.3. *S. enterica* subsp. *diarizonae* isolate with pSASd plasmid did not reduce the lifespan of *C. elegans* more than an isolate without this plasmid

**Figure 5:**
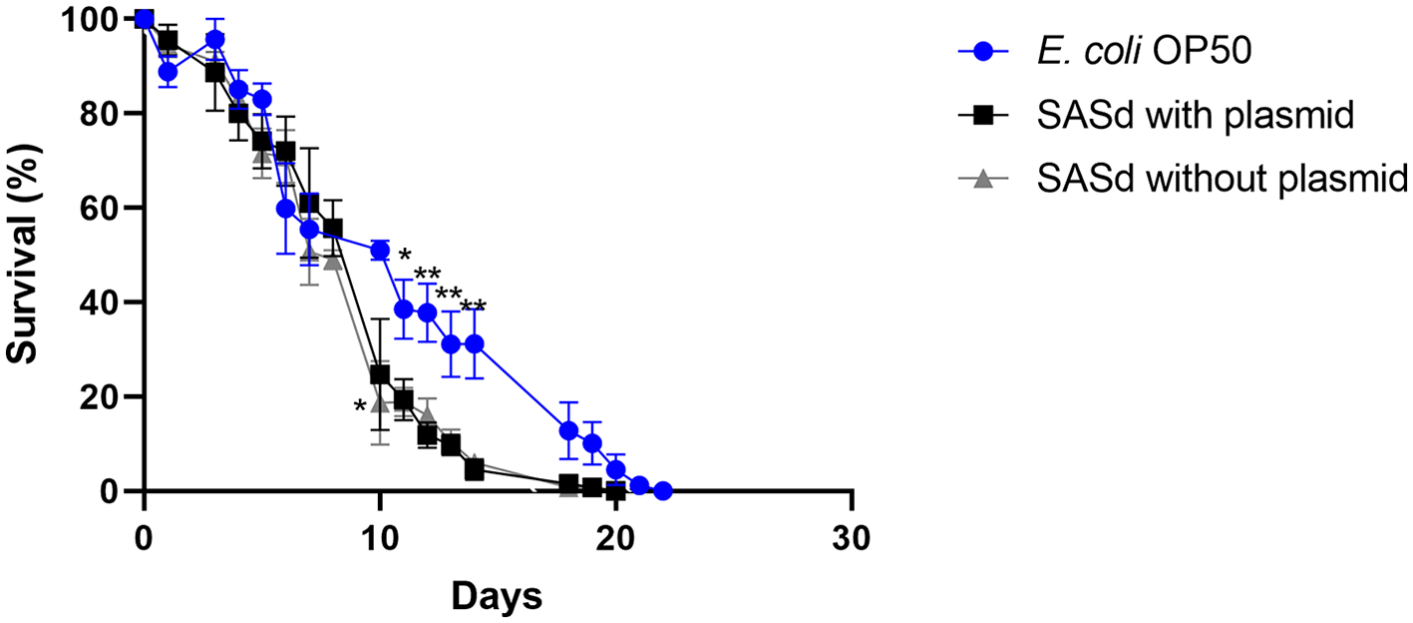
Survival over time of *C. elegans* grown on NGM agar either colonized with the control strain *E. coli* OP50, plasmid-equipped SASd isolate 12-01777-0-S2, or plasmid-free SASd isolate 12-01777-0-S3. Mean with SEM, *n* = 9, ^*^p < 0.05, ^**^p < 0.01.

**Figure 6:**
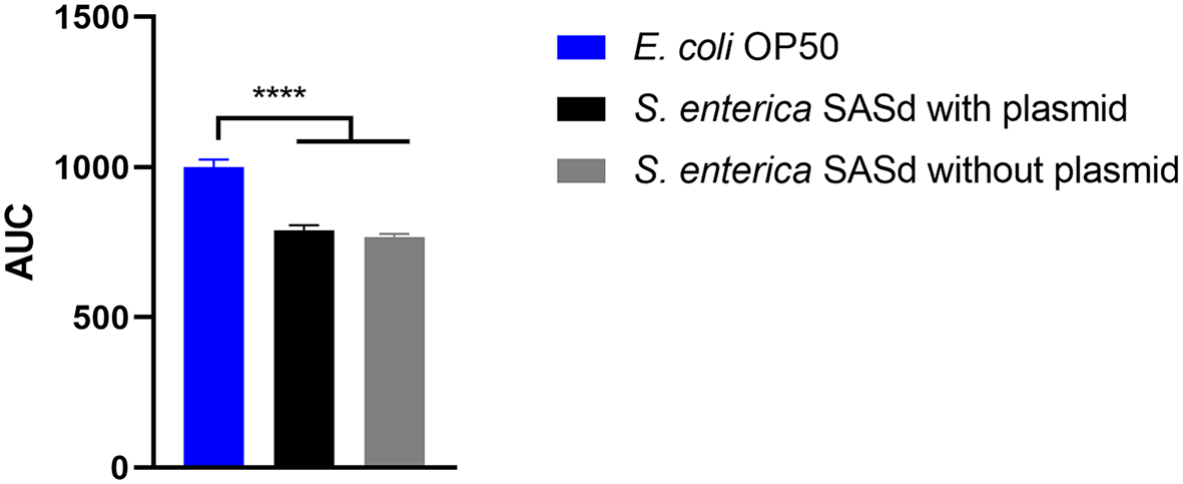
Area under the curve (AUC) of *C. elegans* survival curves. The worms were either fed with *E. coli* OP50 (control), SASd isolate 12-01777-0-S2 (with plasmid), or SASd isolate 12-01777-0-S3 (without plasmid). Mean with SEM, *n* = 9, ^****^p < 0.0001.

Putative virulence factors and the phylogeny of sheep-derived SASd strain 12-01777-0 were investigated previously (Uelze et al., 2021). The strain belongs to the lineage ST432. Two isolates (S2 and S3) were chosen from our strain collection, and their DNA was sequenced using short-read Illumina technology. Analysis of the genomes revealed that S2 and S3 were separate strains with 40 AD between their core genomes, and they additionally differed by the presence (S2) or absence (S3) of a 43 kb plasmid named pSASd containing a T4SS and a toxin/antitoxin system (Uelze et al., 2021). This led to the assumption that isolate 12-01777-0-S2 could be more virulent during the course of infection than isolate 12-01777-0-S3. We applied the *C. elegans* survival assay to both isolates separately. The assay revealed that both isolates reduced the survival rate of *C. elegans* significantly (Gehan-Breslow-Wilcoxon test: *p* = 0.0002) compared to *E. coli* OP50 (Figures 5, 6). We observed clear differences in the rate between day 10 and day 19 after the challenge of up to 27%. However, no differences in the survival rate were detected between worms grown on isolate 12-01777-0-S2 (21% reduction compared to control) and isolate 12-01777-0-S3 without plasmid (23% reduction compared to control, Figures 5, 6).

## 4. Discussion

Approximately 20 years ago, it was shown that broad host range opportunistic pathogens as well as specialized vertebrate pathogens such as *S. enterica* can kill the worm *C. elegans* when it was placed on a lawn of the pathogen (Finlay, 1999; Aballay et al., 2000; Labrousse et al., 2000). The pathogen could proliferate in the *C. elegans* intestine and establish a persistent infection (Aballay et al., 2000). Such a pathogenicity model simplifies scientific studies analyzing the extent of phenotypic traits involved in host-pathogen interactions. It is even conceivable that gene regulatory processes in both the host and the pathogen can be considered for analysis.

In this study, we evaluated the potential of such a *C. elegans* survival assay based on three questions related to the pathogenicity of certain specific *S. enterica* strains. To clarify these questions, animal experiments could also be used and, in the case of vaccination strain Salmovac SE, have been previously reported (Martin et al., 1996b; Theuß et al., 2018).

### 4.1. Wild-type *S*. Enteritidis strain kills *C. elegans* worms faster than the Salmovac SE vaccination strain

In the European Union (EU), laying hens must be vaccinated against *Salmonella*, and live vaccines are commonly used. The excretion of the *Salmonella* vaccine strains can last for weeks. Therefore, in exceptional cases, young laying hen flocks can contaminate their eggs with *Salmonella* vaccine strains. According to Regulation (EC) No. 178/2002, table eggs contaminated with *Salmonella* are assessed as harmful to health and are considered unsafe food. However, no distinction is made between the evidence of vaccine and wild-type *Salmonella* strains on table eggs. It is currently being discussed whether the detection of a *Salmonella* vaccine strain on eggs should be subject to food law measures. The Salmovac SE vaccine strain is an attenuated strain. Attenuation was achieved in two different ways. The strain was derived from the plasmid-free strain Salmonella Enteritidis 6403 PT4. LD50 increases in mouse experiments from <20 to >106 CFU for the plasmid-free Salmonella enterica variant (Martin et al., 1996b). The attenuation was further augmented by introducing adenine and histidine auxotrophy *via* chemical mutagenesis with N-methyl-N-nitro-N-nitrosoguanidine. This auxotrophy reduced the virulence by a factor of ten. However, attenuation based on the histidine and adenine auxotrophy in the *Salmonella* vaccine strain might be compensated in the worm's intestine through direct uptake of amino acids from the gut, as shown in Kern et al. (2016) for Listeria. Nevertheless, the results showed that the combined attenuation of the *Salmonella* vaccine strain was sufficient to restore the lifespan of *C. elegans* to those of worms fed with *E. coli* OP50.

The current results indicate differences in the virulence of *Salmonella enterica* wild strains and the vaccine strain. Nevertheless, it needs to be clarified whether these differences reflect a lack of virulence. It is possible that the observed reduced virulence in nematodes still poses a hazard to vulnerable groups. It is also possible that the reduced virulence observed in nematodes does not apply to humans. We investigated the survival rate of *C. elegans* in the presence of a wild type and in a vaccine *S*. Enteritidis strain (Salmovac SE) derived from the same lineage using the same experimental setup. There have been two types of lethal effects in *C. elegans*. The so-called “fast killing” effect has been linked to *Pseudomonas aeruginosa*, and the nematodes died within 24 h due to intoxication (Finlay, 1999; Tan and Ausubel, 2000). The “slow killing” effect has been related to a more infection-like process and has been found for *Pseudomonas aeruginosa* and *Serratia marcescens* as a second way of killing *C. elegans* (Finlay, 1999; Kurz and Ewbank, 2000). Our investigations clearly showed a “slow killing” effect, which took the pathogens several days to colonize the nematode's gut and kill *C. elegans*. However, it has been reported that *Salmonella enterica* kills *C. elegans* in an even more prolonged process, as observed for the “slow killing” effect of *P. aeruginosa* (Aballay and Ausubel, 2002).

We observed a differentiable virulence between the two strains in the *C. elegans* survival assay. The higher virulent *S*. Enteritidis wild-type strain reduced the lifespan of *C. elegans* significantly as compared to the less virulent vaccine strain. Similar results have been reported by Martin et al. (1996b) using mice. The diminished pathogenicity is a prerequisite for vaccine strains, but a simple and fast test method for this trait was lacking. The *C. elegans* survival assay can be an effective method for discriminating between vaccine and wild-type strains. Another group (Sivamaruthi and Balamurugan, 2014) performed a similar study with a different live vaccine strain (Ty21a) and reported similar results. They further claimed that pre-exposure of *C. elegans* to an *S. enterica* vaccine strain rendered the nematode more resistant to an *S. enterica* wild-type strain infection. These results indicate that the *C. elegans* survival assay is an effective method for pre-screening candidate vaccine strains and identifying vaccine strains among those isolated from laying hens and eggs.

### 4.2. The effect of rough and smooth *S*. Enteritidis strains on the longevity of *C. elegans* did not differ

In total, 60,050 human salmonellosis cases were reported in the EU in 2021, and *Salmonella* Enteritidis was the most commonly isolated (54.6%) serovar (EFSA and ECDC, 2022). Poultry and poultry products are the primary sources of *S*. Enteritidis. Consequently, *S*. Enteritidis is one of the serovars monitored in EU control programs for poultry (Anonymous, 2006, 2008, 2011, 2012). The routine diagnostic determination of *Salmonella* serovars is performed primarily by slide agglutination according to the White-Kauffmann-Le Minor scheme, as outlined in ISO/TR 6579-3:2013. Serotyping is based on the differentiation of the immuno-reactive O-sidechain of the LPS and of two different flagellin(H)antigens. Occasionally, the O-chain reacts non-specifically by classical slide agglutination, leading to non-typeability of the isolates, which are then simply termed “rough”. Rough isolates, however, are not part of the EU *Salmonella* control programs. Therefore, when rough isolates are detected on poultry farms, no action is required, even if the *Salmonella* isolates have been shown by molecular methods to belong to *S*. Enteritidis. In the present study, we demonstrated that there is no difference in virulence between rough and smooth strains of *S*. Enteritidis. Both reduced the lifespan of *C. elegans* in a similar fashion. This finding contrasts literature reports that only a smooth strain of *S. enterica* and not a rough strain leads to the death of germ-free piglets colonized with one or both of these bacteria (Dlabac et al., 1997). However, the applicability to humans is not clear. Rough strains are also known for their increased sensitivity to the immune defense. Therefore, it is unclear whether these strains are non-pathogenic only for fully immune-competent individuals (Lalsiamthara et al., 2018). They may still pose a risk for immunocompromised patients. Therefore, more research is needed to clarify the pathogenic potential of rough and smooth *S. enterica* strains and provide guidance on how to deal with the finding of rough *S. enterica* strains in animals, food, or feed. The *C. elegans* approach might be further helpful in analyzing the pathogenic potential of both variants in more detail, for example, by investigating the upregulation and downregulation of virulence genes within the host during the course of the infection. Nevertheless, our initial results established in this study indicate that both variants should be treated in control measurements in the same way to minimize the entry of these bacteria into the food chain.

### 4.3. The SASd isolate with pSASd plasmid did not reduce the lifespan of *C. elegans* more than an isolate without this plasmid

The SASd isolate is host-adapted to sheep, with a high prevalence in sheep herds worldwide. Infections are usually sub-clinical; however, the serovar has the potential to cause diarrhea, abortions, and chronic proliferative rhinitis. In a previous study (Uelze et al., 2021), we investigated a set of 119 diverse SASd isolates by whole genome sequencing. We found that the serovar was composed of two separate lineages, ST432 and ST439, with different genomic characteristics, of which ST432 was primarily isolated from sheep. We concluded that lineage ST432, in particular, should be considered host-adapted to sheep. In the current study, we investigated two SASd strains of the lineage ST432 in the *C. elegans* survival assay, one with and the other without the pSASd plasmid. Strains of this lineage typically harbor a 43kb large plasmid (pSASd). Although several potential virulence factors are located in this plasmid (Uelze et al., 2021), it does not encode the spv cluster as described for some other virulence plasmids (Gulig et al., 1993; Rotger and Casadesús, 1999). However, the question arises on whether the pSASd plasmid has any influence on the virulence of the strain. We found that the isolates with and without the plasmid both reduced the lifespan of *C. elegans* dramatically, with no difference between them. This indicates that the pSASd plasmid does not have a strong effect on the virulence of the SASd strains under the experimental setup applied in this study. Another group investigated the impact of *S. enterica* strains with and without the pSASd plasmid on macrophages and epithelial cells (Gokulan et al., 2013). They found that plasmid-equipped bacteria showed increased invasion and persistence in those cells and therefore argued that the plasmid enhances the virulence of the *S. enterica* strain. The discrepancy between their and our results can be explained by the fact that *C. elegans* does not have an adaptive immune system or mobile immune cells (Alper et al., 2007; Pukkila-Worley and Ausubel, 2012). Therefore, animal experiments cannot be avoided entirely to elucidate further the role of the pSASd plasmid on the virulence of the strains.

### 4.4. Limits and potential of *C. elegans* assays in studying the virulence and host interaction of bacterial pathogens

In conclusion, the *C. elegans* survival assay cannot replace animal experiments designed to determine differences in the virulence of *Salmonella enterica* strains. Still, it is an effective and relatively easy method for classifying the virulence of different bacterial isolates *in vivo*, despite some limitations. The divergent immune response to pathogens and differences in the course of infection might lead to discrepancies between results obtained with *C. elegans* assays and those from experiments with higher vertebrates. Therefore, we recommend using the described method to pre-screen bacterial strains of interest to select the most promising candidates for further animal experiments. It has been the traditional concept to substitute an animal test only with an alternative test that is fully equivalent. This concept is limited to replacing animal tests for complex questions involving interdependent organ systems. The so-called integrated testing strategies were introduced into toxicology by an ECVAM task force to meet the requirements of complex systems (Blaauboer et al., 1999). The idea behind this strategy is to combine different alternative tests, creating a complexity that should overcome the intrinsic limitation of the stand-alone test systems (Calonie et al., 2022). Currently, the range of alternative systems is very broad, ranging from *in silico* simulations, cell cultures, 3D cell cultures, and organoids to non-vertebrate *in vivo* models. An elaborated combination of these options promises testing approaches with a high human prediction value. Moreover, a much larger pool of validation data is available from comparisons between animal tests and alternative methods (Calonie et al., 2022).

Although *C. elegans* has been widely used to study pathogenicity mechanisms of microorganisms for two decades (Sifri et al., 2005), direct comparisons between animal-based infection models and *C. elegans* infection assays are rare. There are different reasons why such a direct comparison is usually not applied. Unlike other approaches, e.g., in toxicology, investigating infectious pathogenicity is not based on standardized animal tests. The animal tests must be chosen according to the microorganism and the host. For example, mice infected with *Salmonella* Typhimurium did not show any signs of diarrhea and are not suitable as an infectious model for human disease (Santos et al., 2001). Moreover, the investigated endpoints in animal tests and *C. elegans* infection assays are very different, making the comparison difficult. The approach usually applied is to use strains of pathogenic microorganisms with a certain proven virulence or a lack of pathogenicity in animal models or patients. If the clinically observed effects are reflected in similar effects in the *C. elegans* infection assay, the assay can be used to investigate the role of specific pathogen factors. This has been quite successfully applied for pathogenic *E. coli*, where the pathogenicity island locus of enterocyte effacement (lee), which is responsible for the virulence in humans, has also been shown to be correlated with the ability to kill the nematode (Anyanful et al., 2005).

Further development of variants of the *C. elegans* assay should encompass deeper investigations of the host and pathogen, e.g., by omics technologies such as transcriptomics. It is anticipated that the results of such studies will improve the estimation of the pathogenic potential of test organisms. This would lead to reduced dependence on vertebrate experiments, which is in agreement with the 3R (refine, replace, reduce) principle.

## Data availability statement

The datasets presented in this study can be found in online repositories. The names of the repository/repositories and accession number(s) can be found below: https://www.ncbi.nlm.nih.gov/, PRJNA937468 and PRJNA678834.

## Author contributions

WB, BM, and IS designed the study. JF and IS provided the samples and performed pre-analysis and next-generation sequencing. CS and WB performed the experiments with *C*. *elegans*. WB, IS, and MF interpreted the results and wrote the draft manuscript. WB performed the bioinformatics analysis. JF and BM were involved in manuscript revision prior to the submission of the manuscript. All authors contributed to the article and approved the submitted version.
